# Phylosymbiotic Structures of the Microbiota in *Mollitrichosiphum tenuicorpus* (Hemiptera: Aphididae: Greenideinae)

**DOI:** 10.1007/s00248-021-01830-8

**Published:** 2021-08-13

**Authors:** Man Qin, Liyun Jiang, Bakhtiyor R. Kholmatov, Gexia Qiao, Jing Chen

**Affiliations:** 1 Key Laboratory of Zoological Systematics and Evolution, Institute of Zoology, Chinese Academy of Sciences, Beijing, 100101 China; 2 College of Life Sciences, University of Chinese Academy of Sciences, Beijing, 100049 China; 3 Institute of Zoology, Academy of Sciences Republic of Uzbekistan, Bagishamol Str., 232b, Tashkent, 100053 Uzbekistan

**Keywords:** Intraspecific phylosymbiosis, Microbiota variation, Host genetics, Geography, Symbiont diversity

## Abstract

Aphids harbor an array of symbionts that provide hosts with ecological benefits. Microbial community assembly generally varies with respect to aphid species, geography, and host plants. However, the influence of host genetics and ecological factors on shaping intraspecific microbial community structures has not been fully understood. In the present study, using Illumina sequencing of the V3 − V4 hypervariable region of the 16S rRNA gene, we characterized the microbial compositions associated with *Mollitrichosiphum tenuicorpus* from different regions and plants in China. The primary symbiont *Buchnera aphidicola* and the secondary symbiont *Arsenophonus* dominated the microbial flora in *M. tenuicorpus*. Ordination analyses and statistical tests suggested that geography and aphid genetics primarily contributed to the variation in the microbiota of *M. tenuicorpus*. We further confirmed the combined effect of aphid genetics and geography on shaping the structures of symbiont and secondary symbiont communities. Moreover, the significant correlation between aphid genetic divergence and symbiont community dissimilarity provides evidence for intraspecific phylosymbiosis in natural systems. Our study helped to elucidate the eco-evolutionary relationship between symbiont communities and aphids within one given species.

**Supplementary Information:**

The online version contains supplementary material available at 10.1007/s00248-021-01830-8.

## Introduction

The influence of ecological factors and host genetics on animal-associated microbial communities has been well documented [1–3]. Phylosymbiosis occurs when microbial community relationships significantly correlate with the evolutionary history of the host [4, 5]. This pattern does not necessarily presume the vertical inheritance of the entirety or some members of microbial communities. In addition to the codiversification of hosts and some microbes [6], phylosymbiosis may arise from ecological filtering by conserved host traits [7, 8]. Interspecific phylosymbiosis has been substantiated in certain insects, fishes, birds, and mammals [9–12]. At the intraspecific level, Kohl et al. [13] reported the phylosymbiotic relationship between gut microbiota and different geographical populations of *American pikas*. However, investigation of intraspecific phylosymbiosis has rarely been assessed in other animal groups. To completely understand the eco-evolutionary relationship of microbiota and individual host species, intraspecific phylosymbiosis analyses should be performed on more animal groups.

Aphids and their various symbionts provide an excellent model system to study the insect-microbe relationship from ecological and evolutionary perspectives. The microbial communities associated with aphids are typically dominated by symbionts [14–17]. The primary endosymbiont *Buchnera aphidicola*, which resides in specialized bacteriocytes of almost all aphids, provides hosts with essential nutrients lacking in phloem sap diets [18]. *Buchnera* is strictly vertically transmitted from mother to offspring [19] and co-speciates with aphids [20–23]. Aphids also host secondary symbionts that occupy secondary bacteriocytes, sheath cells, or hemocoel [24] and experience vertical and horizontal transmission [25–27]. Most attention has been given to nine secondary symbionts, namely, *Arsenophonus*, *Fukatsuia symbiotica*, *Hamiltonella defensa*, *Regiella insecticola*, *Rickettsia*, *Rickettsiella viridis*, *Serratia symbiotica*, *Spiroplasma*, and *Wolbachia* [25, 28–35]. Secondary symbionts exert diverse mutualistic effects in complex ecological environments, such as resistance to high temperature [36, 37], defense against parasitic wasps and fungal pathogens [38, 39], modification of body colors [34], and enhancement of host plant utilization [40–42].

The factors shaping the symbiont community structures primarily include aphid species [43], characteristics of aphids [17], geography [44], and host plants [16, 45]. The majority of surveys about intraspecific symbiont diversity have focused on the impact of ecological conditions on symbiont infection patterns [46–51]. For example, Tsuchida et al. [52] highlighted that the markedly different infection frequencies of the secondary symbionts in *Acyrthosiphon pisum* were associated with geographical distribution. Regarding the impact of aphid genetic divergence on symbionts, some studies have documented the associations between individual symbionts and hosts, such as the nonrandom presence of *H. defensa* across aphid genetic clusters in *A. pisum* [53] and the divergence of *Buchnera* in different geographical populations of *Schlechtendalia chinensis* [54]. Gauthier et al. [55] demonstrated that the variation in bacterial communities was not related to genetic divergence between biotypes of *A. pisum*. The intraspecific variation in the microbial community needs more exploration across both ecological and aphid genetic contexts.

*Mollitrichosiphum tenuicorpus* (Hemiptera: Aphididae: Greenideinae) is monocious with a holocyclic life cycle. This species feeds on young shoots of plants consisting of *Alnus* (Betulaceae), *Castanospermum* (Fabaceae), *Litsea* (Lauraceae), *Meliosma* (Sabiaceae), and several genera of Fagaceae, such as *Castanea*, *Castanopsis*, *Lithocarpus*, and *Quercus* [56]. *M. tenuicorpus* is distributed in eastern and southeast Asia [56, 57]. Previous studies have demonstrated that *M. tenuicorpus* is divided into three clades [58], which co-segregates with *Buchnera* at the intraspecific level [22]. Qin et al. [43] uncovered the microbial community composition of *M. tenuicorpus*. However, the intraspecific microbiota variation of this species has not been fully characterized to date. *M. tenuicorpus* provides an opportunity to explore the eco-evolutionary relationship between microbiota and insects within one species.

In this study, using Illumina sequencing of the 16S rRNA gene, we characterized the microbial community composition of *M. tenuicorpus* sampled from different plants and regions in China. Moreover, we assessed the effects of aphid genetic divergence, geography, host plant, and environmental conditions on the structures of bacterial, symbiont (incl. *Buchnera* and secondary symbionts), and secondary symbiont communities in the field. To investigate the pattern of intraspecific phylosymbiosis, the correlations between microbiota dissimilarities and aphid genetic divergences were also estimated.

## Materials and Methods

### Aphid Sampling and DNA Extraction

Aphid collection was carried out for seven genera of plants in 12 geographic regions of China. Collection information is shown in Table S1. The samples were frozen at − 20 °C until further processing. All samples were preserved in 75% and 95% ethanol for voucher specimen collections and molecular studies, respectively. Aphid identification was performed using morphological examination and DNA barcoding. All specimens and samples were deposited in the National Zoological Museum of China, Institute of Zoology, Chinese Academy of Sciences, Beijing, China.

A single adult from each sample was obtained for DNA extraction. Aphid individuals were washed with 70% ethanol for 5 min and rinsed with sterile water five times to remove body surface contaminants. Total DNA was extracted from the whole body of each aphid using the DNeasy Blood & Tissue Kit (QIAGEN, Hilden, Germany) according to the manufacturer’s instructions. A negative control substituting the DNA with sterile ultrapure water was prepared in the same way. DNA extracts were PCR-amplified targeting the cytochrome c oxidase subunit I (COI) gene with primers LCO1490 and HCO2198 [59] to verify aphid species and eliminate parasitized samples. Final DNA samples were kept at − 20 °C for further experiments.

### 16S rRNA Gene Amplification and Illumina Sequencing

Primers 341F (5′-CCTAYGGGRBGCASCAG-3′) and 806R (5′-GGACTACNNGGGTATCTAAT-3′) were employed to amplify the V3 − V4 hypervariable region of the 16S rRNA gene. Each 30-μL PCR reaction mixture comprised of 15 μL Phusion High-Fidelity PCR Master Mix (New England Biolabs, Ipswich, MA, USA), 3 μL primers, and 10 μL template DNA. The PCR protocol was as follows: 1 min at 98 °C for initial denaturation; 30 cycles of 10 s at 98 °C for denaturing, 30 s at 50 °C for annealing, and 30 s at 72 °C for elongation; and 5 min at 72 °C for final extension. All samples including negative controls for DNA extraction and amplification were amplified in triplicate.

PCR products were observed on a 2% agarose gel and purified with GeneJET Gel Extraction Kit (Thermo Scientific, Wilmington, DE, USA). Amplicon libraries were prepared with NEBNext Ultra DNA Library Prep Kit (New England Biolabs). Library quality control was performed on Qubit 2.0 Fluorometer (Thermo Scientific) and Agilent Bioanalyzer 2100 system. Finally, sequencing was conducted on an Illumina HiSeq 2500 PE250 platform (Illumina, San Diego, CA, USA).

### Bioinformatic Processing of Sequencing Data

Paired-end reads were merged using FLASH v1.2.7 [60] with a minimum overlap size of 10 bp and an error rate of 10% and assigned to each sample according to their unique barcodes. After filtering and removing chimeras by QIIME v1.9.1 [61], the remaining sequences with ≥ 97% similarity were clustered into the same operational taxonomic units (OTUs) using the UCLUST module. Representative sequences (i.e., the most abundant sequence in OTU clusters) were annotated against the SILVA 128 reference database [62] using the RDP classifier [63] with a 0.80 confidence threshold. Taxonomic classifications were also manually checked by BLAST against GenBank. OTUs with an abundance less than 0.005% were subsequently excluded according to Bokulich et al. [64]. For each sample, we averaged the sequence number across three technical PCR replicates to estimate the abundance of each OTU. To reduce the impact of the uneven sequencing depth on the downstream statistical analyses, the sum of sequence number per sample was rarefied to the minimum value across all samples (53,500 reads) in USEARCH v10.0 [65]. Then, we obtained an OTU count table containing taxonomic definitions and sequence number per sample. We converted OTU count data to relative abundance using the *decostand* function of the R package *vegan* [66]. Finally, the bacterial OTU table was prepared (Table S2a).

### Microbial Community Analyses

To better explore the microbial community structures within *M. tenuicorpus*, OTUs classified as symbionts (incl. *Buchnera* and secondary symbionts) (Table S2b) and secondary symbionts (Table S2c) were screened out, and their relative abundances were calculated via division of the number of sequences assigned to each OTU by the sum of sequences in a given sample. All of the following statistical analyses were performed with bacterial, symbiont, and secondary symbiont data. We grouped all *M. tenuicorpus* samples according to aphid clades, geographic distribution, and host plant. Detailed grouping information is shown in Table S3. A heatmap visualizing the relative abundance of symbiont OTUs was generated using the *pheatmap* function in the R package *pheatmap* [67]. The maximum-likelihood trees showing the relatedness of symbionts and *M. tenuicorpus* aphids were constructed separately using RAxML v8.2.7 [68] (detailed methods are provided in the Electronic Supplementary Methods). Statistical inference was performed on all groups and groups with a sample size ≥ 3, except Mantel tests, Procrustes analyses, and redundancy analyses. Samples with ambiguous host plant information were excluded from analyses.

Based on OTU tables, Shannon and Simpson indices quantifying alpha diversity were assessed using the *diversity* function in *vegan*. Because the alpha diversity data were not normally distributed (Shapiro–Wilk test, *P* < 0.05), nonparametric Kruskal–Wallis tests were conducted to compare the microbiota variation across different groups of aphid clades, geographic distribution, and host plant.

Then, beta diversity, including the Jaccard presence/absence metric and Bray–Curtis relative abundance metric, was calculated using the *vegdist* function of *vegan*. We used unconstrained nonmetric multidimensional scaling (NMDS) and constrained principal coordinate analysis (cPCoA) to visualize the patterns from beta diversity data. NMDS was assessed with the *metaMDS* function of *vegan* (stress values < 0.05 indicate excellent representation). CPCoA was performed using the *capscal*e and *anova.cca* functions in *vegan*. The significance of differences in microbial community structures was examined through analysis of similarities (ANOSIM; *anosim* function) and permutational multivariate analysis of variance (PERMANOVA; *adonis* function) with 9999 permutations in *vegan*. Both statistical tests were calculated based on Jaccard and Bray–Curtis distances, which generate a *P* value and a sample statistic (i.e., *R* of ANOSIM and *R*^*2*^ value of PERMANOVA). An *R* value between 0 and 1 represents the dissimilarity of community structures among groups, and the *R*^*2*^ value measures the degree of difference between two groups.

To further investigate the impact of aphid genetic divergence or geography on microbiota dissimilarities, partial Mantel test using matrices of aphid genetic divergence and beta diversity (Jaccard and Bray–Curtis distances) was performed. Aphid genetic divergences were evaluated by *p* distances between pairs of cytochrome c oxidase subunit I (COI) sequences in MEGA v7.0 [69]. Geographic distances were calculated in Geographic Distance Matrix Generator v1.2.3 [70]. The partial Mantel test is commonly used to examine the relationship between two matrices (e.g., microbial beta diversity and geographic distances) while holding another (e.g., genetic distances) constant. Analyses were implemented using the *mantel* function of the *ecodist* package with 9999 permutations [71]. We also performed multiple regression on distance matrices (*MRM* function of *ecodist*) to assess the combined effect of aphid genetic divergence and geography in shaping the microbial community structures [72]. Moreover, Mantel test (*mantel* function of *vegan*) and Procrustes *(procrustes* and *protest* function of *vegan*) analysis were conducted to investigate the intraspecific phylosymbiosis in *M. tenuicorpus* using the distances of aphid genetic and beta diversity. The Mantel test is a commonly used approach to estimate the relationship between two matrices. Procrustes analysis is more powerful [73], in which *M*^*2*^ varies from 0 (complete incongruence) to 1 (complete congruence).

Finally, to assess the importance of environmental factors in explaining the variation in microbial communities associated with *M. tenuicorpus*, redundancy analyses (RDA) were implemented on OTU tables. Environmental variables were generated from the “WorldClim” dataset using the *getData* function in the *raster* package and logarithmically transformed for normalization. We manually removed collinear environmental variables (function *vif.cca* of *vegan*) and obtained the maximum adjusted *R*^2^. The maximum temperature of the warmest month (Bio5) and annual precipitation (Bio12), latitude, and altitude were extracted as predictor variables. Next, the RDA model was applied using the *rda* function in *vegan* to study the relationship between the microbiota and these screened environmental variables.

## Results

### Microbial Community Profiling

We obtained 1,469,838 reads after quality control, with a mean of 56,532 reads per sample. The sequences were assigned into 106 OTUs, which belonged to 46 genera, 35 families, 25 orders, 14 classes, and 7 phyla of bacteria. Proteobacteria (average relative abundance across all samples, 98.85%) was the most dominant phylum of the microbial composition associated with *M. tenuicorpus*. At the class level, Gammaproteobacteria (96.66%) represented the most commonly classified bacteria. Enterobacteriales (96.28%) was the most abundant order, followed by Rickettsiales (1.84%). Enterobacteriaceae (96.26%) and Anaplasmataceae (1.41%) were common, and other bacterial families accounted for less than 0.50% (Table S4). Among the top 10 genera, the relative abundances of *Buchnera aphidicola* (83.62%), *Arsenophonus* (10.52%), and *Wolbachia* (1.41%) were greater than 1%. The alpha diversity estimates of bacterial communities across *M. tenuicorpus* samples ranged from 0.117 to 0.799 for the Shannon index and from 0.077 to 0.684 for the Simpson index (Table S5).

**Fig. 1:**
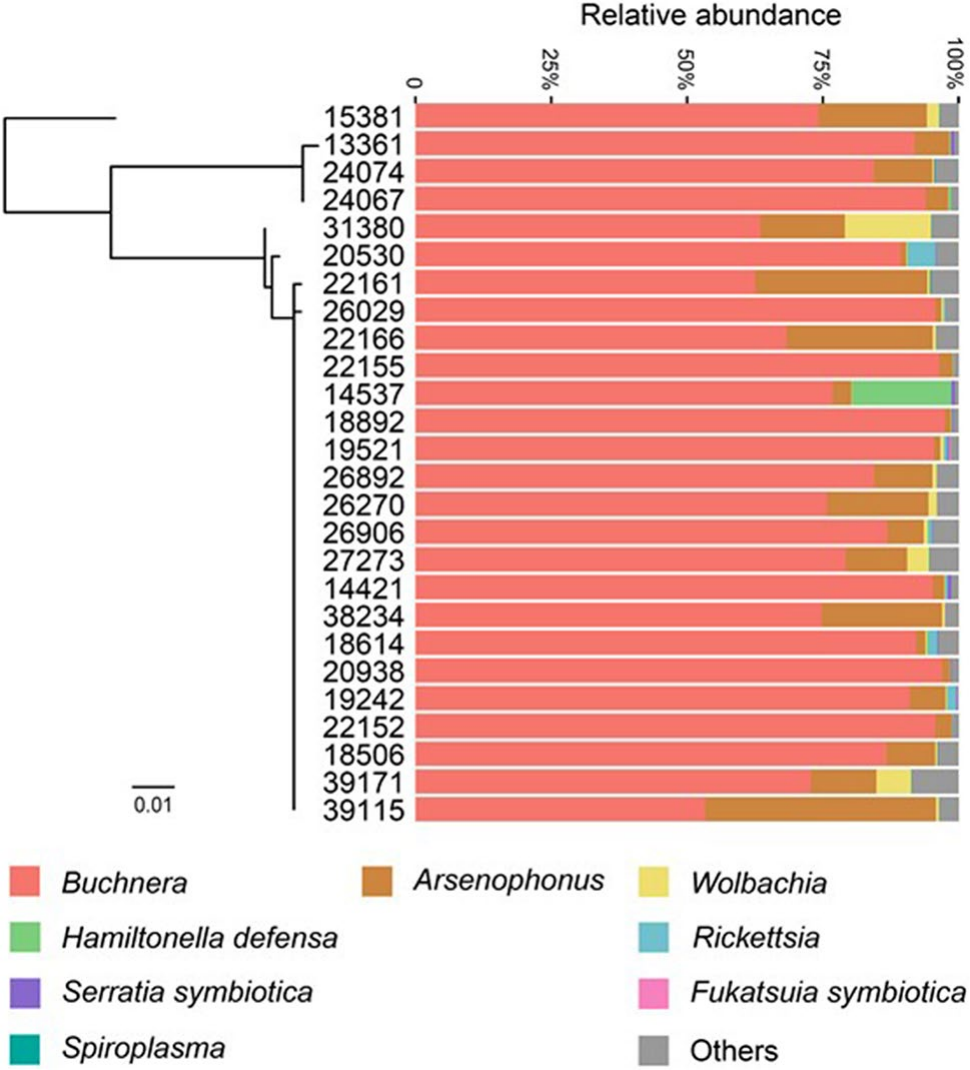
Microbial community composition associated with *Mollitrichosiphum tenuicorpus* across all samples. The maximum-likelihood tree depicts the phylogenetic relationships between aphid samples

Each sample examined in this study simultaneously harbored 4–7 symbionts (Fig. 1). The primary endosymbiont *Buchnera* and secondary symbionts *Arsenophonus* and *Wolbachia* were detected in all samples (infection frequency, 26/26). In addition, *M. tenuicorpus* was infected with five other kinds of aphid secondary symbionts in which the relative abundances were low, including *Hamiltonella defensa* (11/26, 0.77%), *Rickettsia* (14/26, 0.41%), *Serratia symbiotica* (22/26, 0.13%), *Spiroplasma* (12/26, 0.04%), and *Fukatsuia symbiotica* (3/26, 0.02%). The alpha diversity estimates of symbiont communities across *M. tenuicorpus* samples ranged from 0.085 to 0.695 for the Shannon index and from 0.061 to 0.661 for the Simpson index (Table S5). After excluding the primary endosymbiont *Buchnera*, the alpha diversity estimates of secondary symbiont communities ranged from 0.184 to 0.923 for the Shannon index and from 0.143 to 0.808 for the Simpson index (Table S5).

**Fig. 2:**
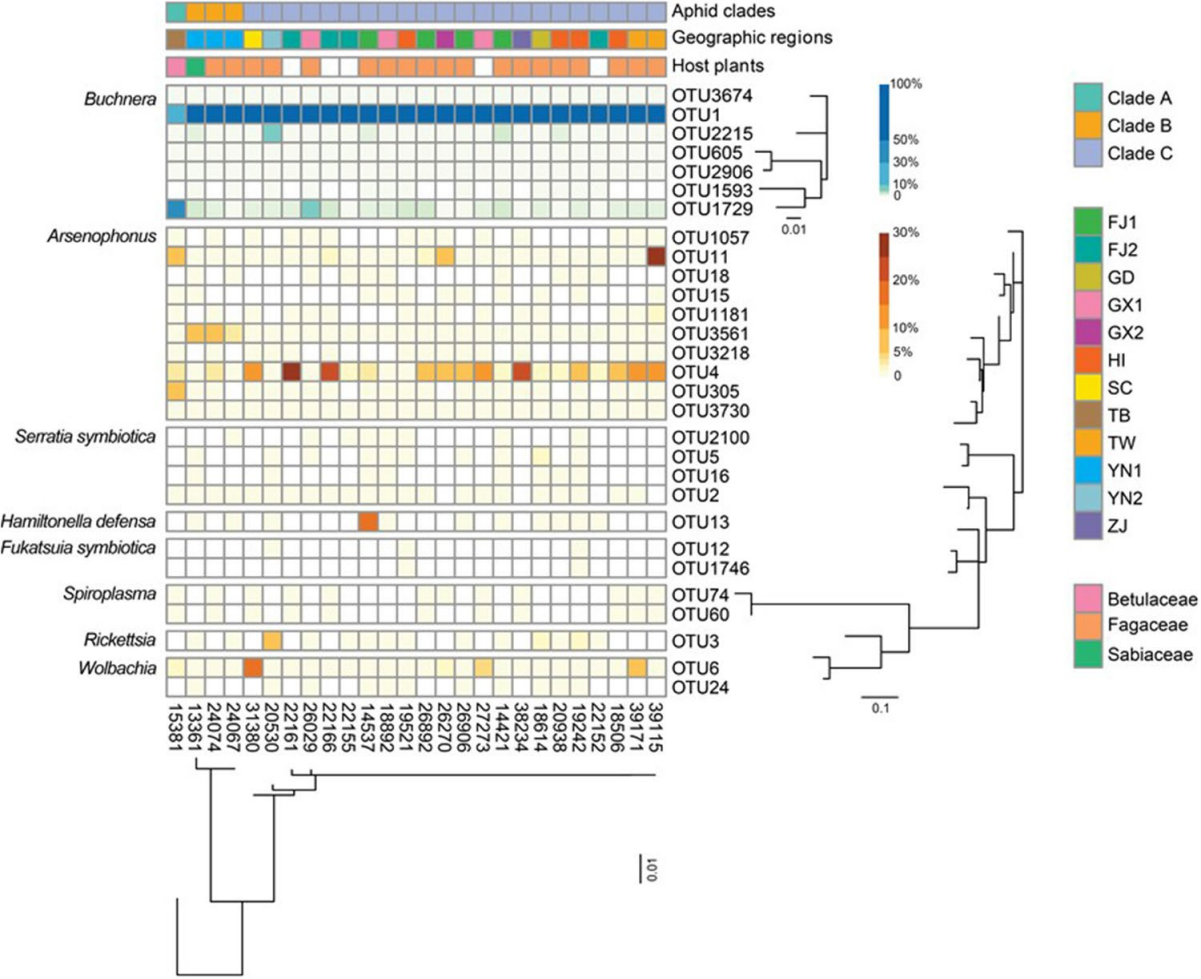
Heatmap representing the distribution and relative abundances of symbiont OTUs among *Mollitrichosiphum tenuicorpus*. The maximum-likelihood trees display the phylogenetic relationships of *Buchnera* OTUs, secondary symbiont OTUs, and *Mollitrichosiphum tenuicorpus*, respectively. White groups indicate ambiguous information on the host plant

At the OTU level, OTU1 of *Buchnera* predominated in the most samples with a relative abundance of 81.66%, except for a sample from Tibet in which OTU1729 (48.70%) and OTU1 (25.45%) were most abundant (Fig. 2). Regarding the secondary symbionts, the predominant OTUs of *Arsenophonus* differed among the different aphid clades in *M. tenuicorpus*. The secondary symbiont composition of the sample collected from Tibet (sample ID: 15381) was dominated by OTU11 (9.49%) and OTU305 (5.61%) belonging to *Arsenophonus*. The most abundant secondary symbiont OTU of samples from northwestern Yunnan Province (sample ID: 13361, 24074 and 24067) was OTU3561 of *Arsenophonus*, in which the relative abundance ranged from 3.01 to 6.16%. OTU4 of *Arsenophonus* dominated the secondary symbiont communities of most samples widely distributed in southern China, with an average relative abundance of 7.92%.

### Microbial Community Signatures of *Mollitrichosiphum tenuicorpus*

Kruskal–Wallis tests based on alpha diversity indices did not reveal a significant effect of aphid genetic divergence (Shannon index, *P* = 0.213–0.616; Simpson index, *P* = 0.192–0.676) or host plants (Shannon, *P* = 0.155–0.624; Simpson, *P* = 0.163–0.566) on the bacterial, symbiont, and secondary symbiont communities. No significant variation in microbial communities was found among geographic regions (Shannon, *P* = 0.057–0.837; Simpson, *P* = 0.113–0.875), except for a significant variation in the secondary symbiont community based on the Simpson index (*n* ≥ 3, *P* = 0.036).

**Fig. 3:**
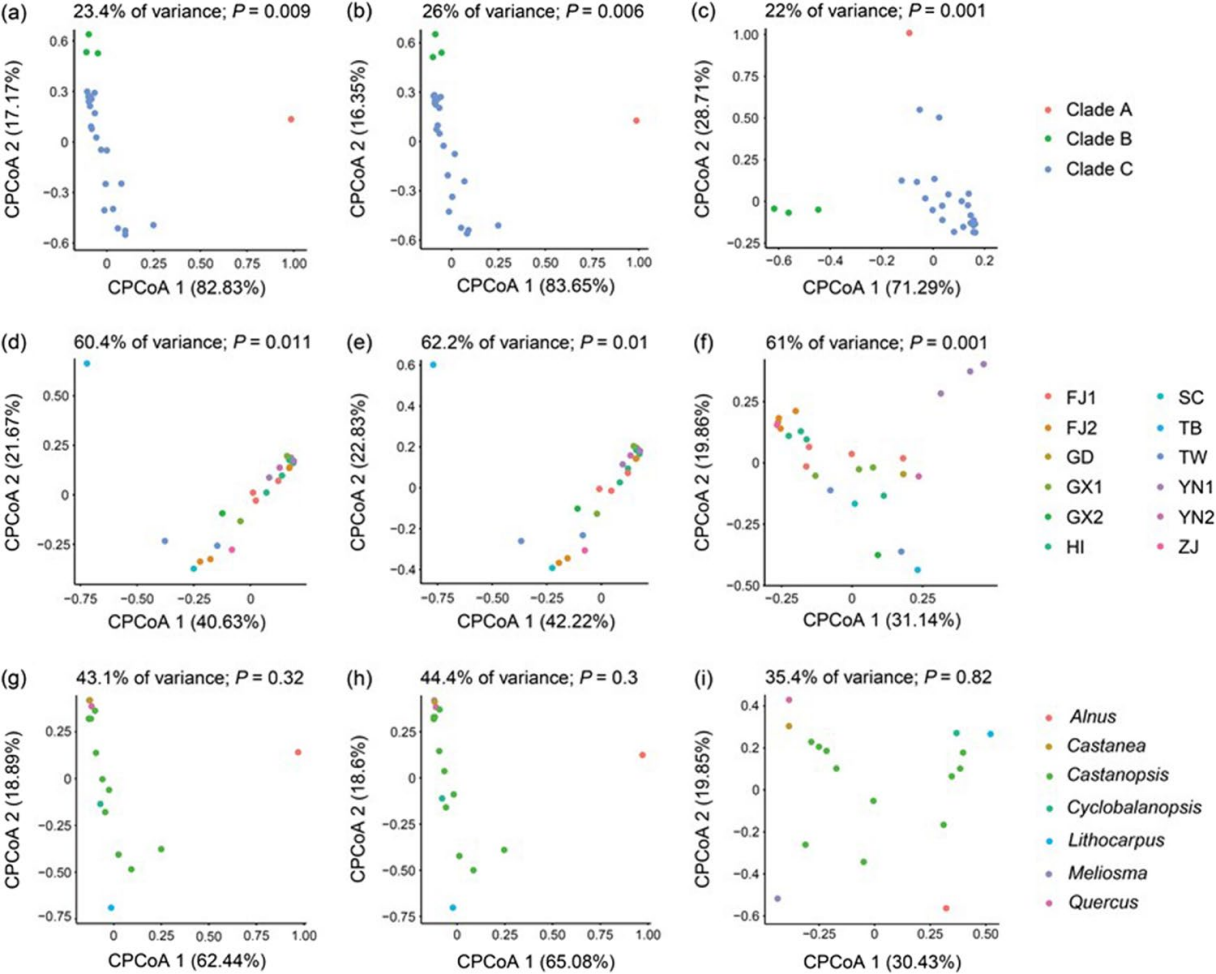
Constrained principal coordinate analysis (cPCoA) plots based on Bray–Curtis distances of bacterial (a, d, g), symbiont (b, e, h), and secondary symbiont (c, f, i) communities (*n* ≥ 1). Plots are structured by aphid clades (a–c), geographic region (d–f), and host plant (g–i). The abbreviations are given in Table S3

There was no recognizable clustering of samples structured by aphid clades, geographic regions, or host plants in unconstrained NMDS plots (Fig. S1–S3) using all types of beta diversity data. Conversely, constrained PCoA (cPCoA) analyses based on Jaccard and Bray–Curtis distances using all samples showed a significant beta-diversity pattern. CPCoA plots displayed significant clustering constrained by aphid clades (*P* = 0.001–0.013; Fig. 3a–c and Fig. S4a–c) and geographic regions (*P* = 0.001–0.011; Fig. 3d–f and Fig. S4d–f) in the bacterial, symbiont, and secondary symbiont communities. The overall variance in the data explained by geographic regions (57.1–62.2% of variance) was greater than that explained by aphid clades (18.2–26% of variance). Moreover, cPCoA analyses did not reveal a meaningful microbial community structure constrained by host plants (35.4–44.4% of variance, *P* = 0.3–0.87; Fig. 3g–i and Fig. S4g–i). Regarding beta diversity with a sample size ≥ 3, only cPCoA analyses of geographic groups were performed, as the data constrained by aphid clades and host plant were insufficient. Additionally, the cPCoA analyses merely uncovered a significant pattern of secondary symbiont community among geographic regions (*P* = 0.001; 40% of variance for Jaccard distances; 44.1% of variance for Bray–Curtis distances; Fig. S5c, f).

ANOSIM did not detect a significant impact of aphid genetic divergence (*R* =  − 0.153 − 0.181, *P* = 0.180–0.741) or geography (*R* =  − 0.163 − 0.045, *P* = 0.271 − 0.935) on shaping the structures of bacterial and symbiont communities (Table 1). Nonetheless, the variation in secondary symbiont communities was usually significant among samples grouped by aphid clades (*R* = 0.627 − 0.640, *P* = 0.003 − 0.008) and geographic regions (n ≥ 3, *R* = 0.388, *P* < 0.001). PERMANOVA corroborated that the secondary symbiont communities significantly differed among aphid clades (*R* = 0.196 − 0.303, *P* < 0.001) and geographic regions (*R* = 0.484 − 0.535, *P* < 0.001) (Table 1). The *R*^2^ values further suggested a greater contribution of geography than aphid genetic divergence. PERMANOVA also indicates a significant dissimilarity among samples in some datasets from different aphid clades (*n* ≥ 1, *R*^*2*^ = 0.323 − 0. 492, *P* = 0.008 − 0.009) and geographic regions (*n* ≥ 3, *R*^*2*^ = 0.262 − 0.286, *P* = 0.012 − 0.026) in bacterial and symbiont communities. Moreover, neither ANOSIM (*R* =  − 0.019 − 0.038, *P* = 0.401 − 0.550) nor PERMANOVA (*R* = 0.374 − 0.630, *P* = 0.242 − 0.506) revealed a significant effect of host plant.

**Table 1:** Results of ANOSIM and PERMANOVA based on Jaccard and Bray–Curtis distances

Beta diversity distance	Microbial community	Sample size	Aphid clades		Geographic region		Host plant	
			ANOSIM (*R*, *P*)	PERMANOVA (*R*^2^, *P*)	ANOSIM (*R*, *P*)	PERMANOVA (*R*^2^, *P*)	ANOSIM (*R*, *P*)	PERMANOVA (*R*^2^, *P*)
Jaccard	Bacteria	*n* ≥ 1	0.164, 0.195	0.323, *0.009*	− 0.163, 0.933	0.326, 0.899	0.008, 0.456	0.508, 0.271
		*n* ≥ 3	− 0.153, 0.734	0.055, 0.222	0.016, 0.379	0.260, 0.307	–	–
	Symbionts	*n* ≥ 1	0.181, 0.180	0.363, *0.008*	− 0.163, 0.935	0.314, 0.898	0.038, 0.401	0.533, 0.248
		*n* ≥ 3	− 0.129, 0.665	0.059, 0.206	0.045, 0.274	0.272, 0.260	–	–
	Secondary symbionts	*n* ≥ 1	0.627, *0.003*	0.252, < *0.001*	− 0.073, 0.723	0.435, 0.521	− 0.019, 0.550	0.375, 0.472
		*n* ≥ 3	0.640, *0.008*	0.196, < *0.001*	0.388, < *0.001*	0.484, < *0.001*	–	–
Bray–Curtis	Bacteria	*n* ≥ 1	0.164, 0.186	0.444, *0.008*	− 0.163, 0.929	0.301, 0.882	0.008, 0.459	0.602, 0.264
		*n* ≥ 3	− 0.153, 0.741	0.057, 0.236	0.016, 0.374	0.258, 0.330	–	–
	Symbionts	*n* ≥ 1	0.181, 0.180	0.492, *0.009*	− 0.163, 0.932	0.292, 0.882	0.038, 0.409	0.630, 0.242
		*n* ≥ 3	− 0.129, 0.669	0.060, 0.218	0.045, 0.271	0.268, 0.294	–	–
	Secondary symbionts	*n* ≥ 1	0.627, *0.003*	0.303, < *0.001*	− 0.073, 0.728	0.427, 0.559	− 0.019, 0.550	0.374, 0.506
		*n* ≥ 3	0.640, *0.006*	0.244, < *0.001*	0.388, < *0.001*	0.535, < *0.001*	–	–

Statistically significant *P* values (*P* < 0.05) are highlighted in italics

Based on partial Mantel tests, we did not detect a significant correlation between aphid genetic divergence and microbial profiles comprising the bacterial, symbiont, and secondary symbiont communities (*r* = 0.162 − 0.358, *P* = 0.844 − 0.993; Table S6) after removing the effect of geography. The microbial community structures were not significantly related to geography when controlling for the effect of aphid genetic divergence (*r* = 0.097 − 0.121, *P* = 0.894 − 0.947; Table S6). However, multiple regression on distance matrices revealed the significant combined effect of aphid genetic divergence and geography on symbiont (Bray–Curtis distance, *R*^2^ = 0.142, *P* = 0.048) and secondary symbiont communities (Jaccard distance, *R*^2^ = 0.229, *P* = 0.001; Bray–Curtis distance, *R*^2^ = 0. 250, *P* = 0.002) (Table 2). We further found a significant correlation between the distances of aphid genetic divergence and geography using the Mantel test (*r* = 0.6238, *P* < 0.001).

**Table 2:** Relationships between microbial communities and the combined effect of aphid genetic divergence and geography revealed by multiple regression on distance matrices

Microbial community	Beta diversity distance	*R*^2^	*P*
Bacteria	Jaccard	0.099	0.060
	Bray–Curtis	0.132	0.052
Symbionts	Jaccard	0.109	0.055
	Bray–Curtis	0.142	*0.048*
Secondary symbionts	Jaccard	0.229	*0.001*
	Bray–Curtis	0.250	*0.002*

Statistically significant *P* values (*P* < 0.05) are highlighted in italics

**Fig. 4:**
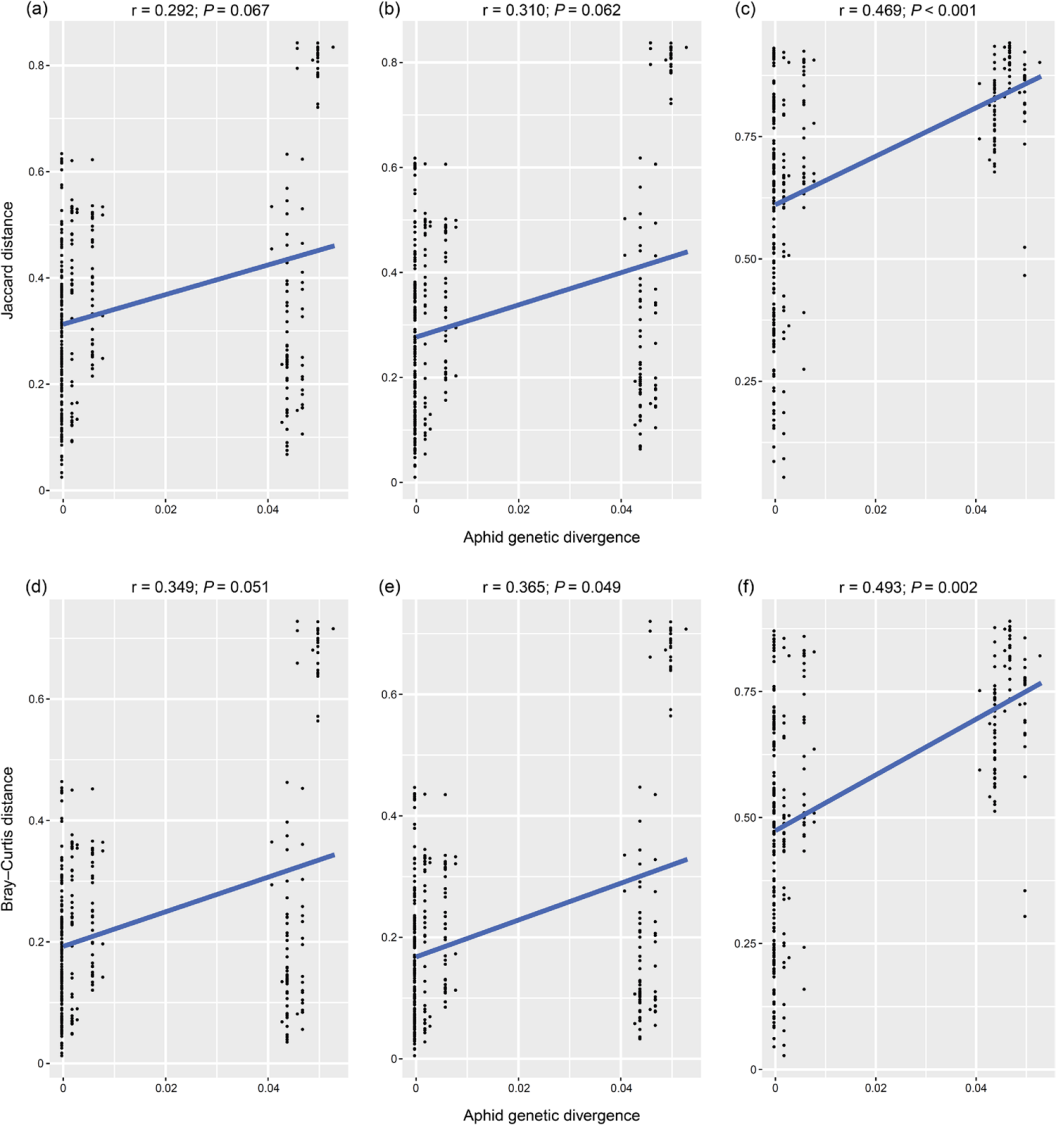
Correlations between microbial beta diversity and aphid genetic distances estimated by Mantel tests in bacterial (**a**, **d**), symbiont (**b**, **e**), and secondary symbiont (**c**, **f**) communities. Microbial beta diversity was assessed by Jaccard (**a**–**c**) and Bray–Curtis (**d**–**f**) distances

**Fig. 5:**
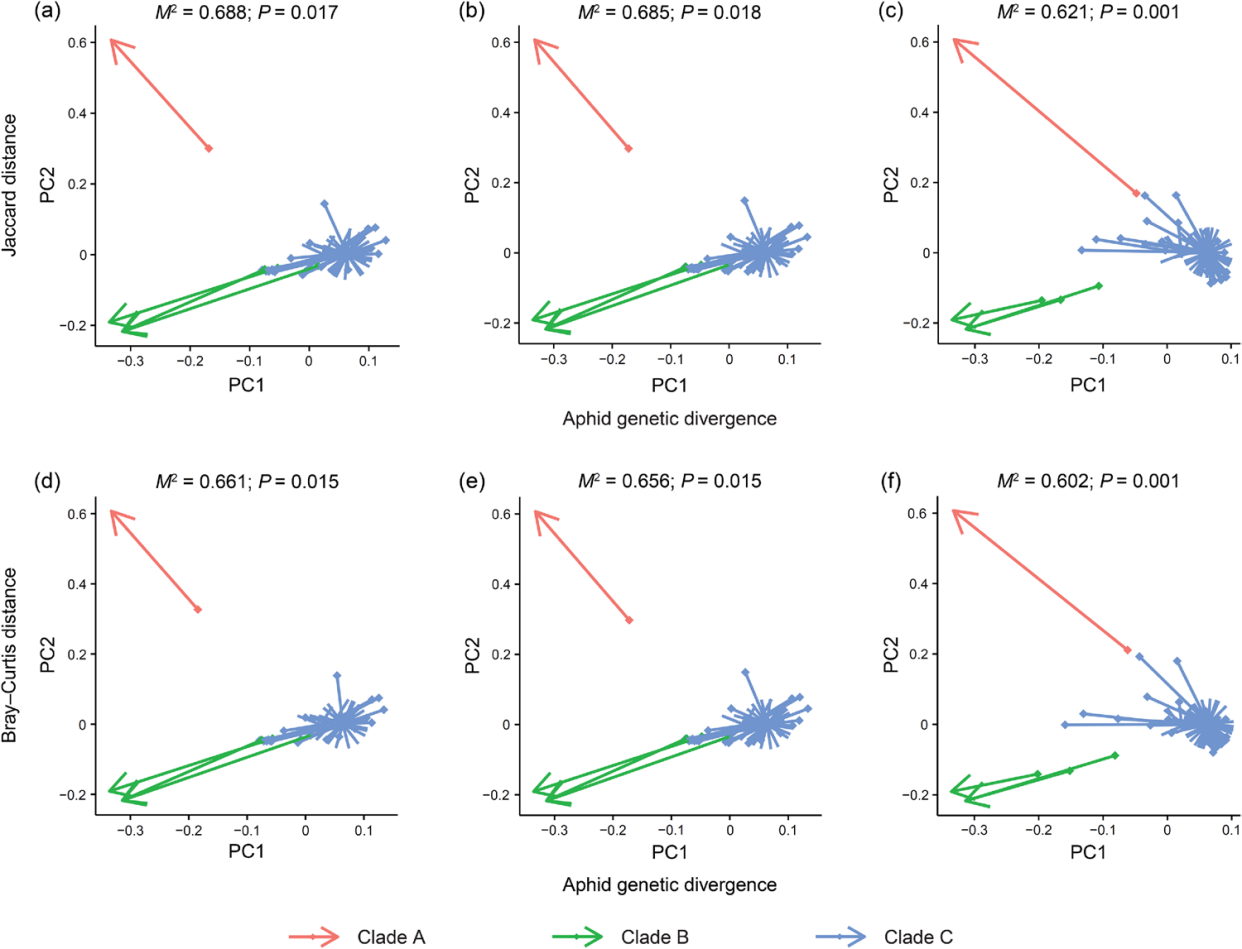
Procrustean superimpositions for PCA-scaled aphid genetic distances vs. variations in bacterial (**a**, **d**), symbiont (**b**, **e**), and secondary symbiont (**c**, **f**) communities. Jaccard (**a**–**c**) and Bray–Curtis (**d**–**f**) distances were used to estimate the microbiota variations

Based on Mantel tests, we observed a significant positive correlation between aphid genetic divergence and symbiont community dissimilarities (Bray–Curtis distances, *r* = 0.365, *P* = 0.049; Fig. 4e). The same linkage between aphid genetic divergence and secondary symbiont communities was also found (Jaccard distance, *r* = 0.469, *P* < 0.001; Bray–Curtis distance, *r* = 0.493, *P* = 0.002; Fig. 4c, f). Using Procrustes analyses, a significantly strong correlation between aphid genetic divergence and microbial communities was confirmed in the bacterial, symbiont, and secondary symbiont communities (*M*^*2*^ = 0.602 − 0.688, *P* = 0.001 − 0.018; Fig. 5).

### Influence of Environmental Factors on Microbiota

As indicated by RDA, four environmental variables (Bio5, Bio12, latitude, and altitude) were significantly related to the bacterial community composition (*R*^2^ = 0.131, *P* = 0.045). In the RDA ordination plot, axis 1 and axis 2 explained 10.52% and 1.26%, respectively, of the variance in the relationship between the environmental variables and bacterial communities (Fig. S6). However, we did not detect a meaningful impact of the screened environmental factors on symbiont (*R*^2^ = 0.102, *P* = 0.077) and secondary symbiont communities (*R*^2^ = 0.110, *P* = 0.065).

## Discussion

### Symbiont Diversity of *Mollitrichosiphum tenuicorpus*

Aphid symbionts dominated the microbial community composition of *M. tenuicorpus*, among which *Buchnera* was the most abundant bacteria in all examined samples. This confirms the essential role of *Buchnera* in aphid survival and reproduction [74, 75]. In addition, *M. tenuicorpus* simultaneously harbored three to six types of secondary symbionts per sample. The frequent coinfection pattern in the present study substantiated the multiple infections of secondary symbionts within one aphid host reported in *Mollitrichosiphum* aphids [43].

*Arsenophonus* and *Wolbachia* were harbored by all examined *M. tenuicorpus* samples with high relative abundance, which suggested their ubiquity in aphids [14, 16, 17, 76]. *Arsenophonus* dominated the secondary symbiont community composition of *M. tenuicorpus* samples with the highest infection frequency and relative abundance. Moreover, we observed a high diversity of *Arsenophonus*, which was represented by ten types of OTUs in *M. tenuicorpus*. Previous studies have demonstrated that *Arsenophonus* can provide aphids with general fitness benefits [77] and facilitate specialization on a novel host plant [42, 78]. *Wolbachia* plays a role in manipulating the reproduction [79] of numerous terrestrial arthropods [80]. However, the precise impact of *Wolbachia* on aphids has not been explored. Further investigations are needed to illustrate the exact effects of *Arsenophonus* and *Wolbachia* on *M. tenuicorpus* aphids.

The other five secondary symbionts, namely, *H. defensa*, *Rickettsia*, *S. symbiotica*, *Spiroplasma*, and *F. symbiotica*, presented low relative abundances in *M. tenuicorpus*. *Spiroplasma* was detected for the first time in *Mollitrichosiphum* aphids, although its relative abundance was low. *Spiroplasma* has been reported in few aphid groups, including *Aphis gossypii* [16], *Myzus persicae* [81], *Aphis citricidus* [82], and some species in Eriosomatinae [45]. The prevalence of *Spiroplasma* may be underestimated in other aphids due to the low titers. In addition, we found an infection pattern of *F. symbiotica* with low prevalence and relative abundance in *M. tenuicorpus*, which is different from other aphid species in *Mollitrichosiphum* [43]. Only one sample harbored *H. defensa* with a relative abundance of 18.22%. After removing this sample, the relative abundance of *H. defensa* across all examined *M. tenuicorpus* aphids was low (0.07%). *M. tenuicorpus* aphids have extremely long siphunculi and can efficiently emit alarm pheromones [83] to reduce the risk of predation. Aphids usually do not carry secondary symbionts, providing the benefits that they have already conferred from ecological traits [49]. This may explain the low relative abundance of defensive secondary symbiont *H. defensa* in *M. tenuicorpus*.

### Factors Determining Microbiota Variation within *Mollitrichosiphum tenuicorpus*

The influence of host plants on microbial communities has been reported within one aphid species [16, 55, 82]. Nevertheless, some studies have demonstrated that geographical distribution plays a more important role than host plant in determining the microbial flora within one aphid species [46, 84]. In the present study, the effect of host plant on microbial community structures associated with *M. tenuicorpus* was not significant. Our results revealed the greatest contribution of geography in shaping the structures of bacterial, symbiont, and secondary symbiont communities in *M. tenuicorpus*. The geographic variability of microbiota may arise from local environmental conditions [85]. However, we found only a weakly significant association between environmental factors and bacterial communities. The nonsignificant relationships between environmental variables and the structures of symbiont and secondary symbiont communities rule out the impact of abiotic features among different geographical regions.

Notably, we detected a significant combined effect of aphid genetic divergence and geography on the symbiont and secondary symbiont communities, while their separate effects were not significant using the partial Mantel test. The Mantel test confirmed the significant correlation between aphid genetic divergence and geographic distances in the present study. Overall, our results suggest that the structures of symbiont and secondary symbiont communities are determined by the combination of aphid genetic divergence and geography.

### Intraspecific Phylosymbiosis in *Mollitrichosiphum tenuicorpus*

Procrustes analyses showed that the genetic divergence of *M. tenuicorpus* significantly related to the microbial profiles of bacterial, symbiont, and secondary symbiont communities using all types of data. We also detected the phylogenetic structures of symbiont and secondary symbiont communities with Mantel tests. Although there was little inconsistency in the results under different analysis methods and beta diversity distance metrics, the phylosymbiotic signals within one aphid species were uncovered in the symbiont and secondary symbiont communities.

Significant positive correlations between aphid relatedness and microbiota dissimilarities have been reported in aphids at the interspecies level [43, 86]. Various factors can lend to the phylogenetically structured microbiota, such as host filtering of environmental microbes [7], regulation of host immune system [87], host phylogeny-related diet preference [88], and host-microbe codiversification [6]. We detected a nonsignificant impact of environmental factors and host plants on the symbiont communities in *M. tenuicorpus*. Considering the similarities of host traits within *M. tenuicorpus*, it is less likely that the phylosymbiotic structures result from ecological filtering by aphid traits. Alternatively, some specific microbes serving as keystone or hub taxa can determine the composition of the whole microbiota via microbe-microbe interactions [2, 89]. Codiversification of hub microbes and hosts can lend to the pattern of phylosymbiosis. We suggest that the phylosymbiotic structures in symbiont communities associated with *M. tenuicorpus* are driven by the codiversification of aphids and predominant symbionts (i.e., the primary endosymbiont *Buchnera* and the secondary symbiont *Arsenophonus*).

The co-segregation between three clades of *M. tenuicorpus* and maternally inherited *Buchnera* has been substantiated by Liu et al. [22]. The divergence of the clade within *M. tenuicorpus* sampled from Tibet occurred earlier than that of other clades [90]. Despite the limitation of phylogenetic signals in the V3 − V4 hypervariable region of the 16S rRNA gene, the diversification of predominant OTUs belonging to *Buchnera* corresponded to host aphids in the present study. In addition, the variation in dominant OTUs of the secondary symbiont *Arsenophonus* was generally congruent with aphid genetic divergence, which indicates host-symbiont codiversification within *M. tenuicorpus*. However, further investigations with additional data are required to elucidate the shared diversification history between *M. tenuicorpus* and *Arsenophonus*.

## Conclusions

Assessing the factors determining microbial communities is crucial for understanding of host-microbiome associations. We identified the microbial composition dominated by symbionts within one aphid species, *Mollitrichosiphum tenuicorpus*. The combined impact of aphid genetic divergence and geography was uncovered in the symbiont community profiles. Moreover, we provided evidence of intraspecific phylosymbiosis based on the significant correlation between *M. tenuicorpus* and symbiont flora. We highlighted the role of codiversification in shaping the phylosymbiotic pattern of *M. tenuicorpus*, paving the way for further investigations of aphid-symbiont interactions in natural populations.

## Supplementary Information

Below is the link to the electronic supplementary material.

Supplementary file1 (DOCX 3788 KB)

Supplementary file2 (XLS 71 KB)

## Data Availability

All COI sequences were deposited in GenBank under accession numbers MZ073749–MZ073754. Raw 16S rRNA gene amplicon reads were submitted in the NCBI Sequence Read Archive under BioProject accession number PRJNA726742.
