# Supplementary material for: Phylosymbiotic Structures of the Microbiota in Mollitrichosiphum tenuicorpus (Hemiptera: Aphididae: Greenideinae)
Source: Microb Ecol. 2021 Aug 13;84(1):227–39. doi: 10.1007/s00248-021-01830-8 (PMC9250915; doi:10.1007/s00248-021-01830-8)
Supplement: Supplementary file 1 — Supplementary file1 (DOCX 3788 KB) [file 248_2021_1830_MOESM1_ESM.docx]

**Electronic Supplementary Material**

**Phylosymbiotic structures of the microbiota in *Mollitrichosiphum tenuicorpus* (Hemiptera: Aphididae: Greenideinae)**

Man Qin^1,3^, Liyun Jiang^1^, Kholmatov Bakhtiyor Rustamovich^2^, Gexia Qiao^1,3*^, Jing Chen^1*^

^1^ Key Laboratory of Zoological Systematics and Evolution, Institute of Zoology, Chinese Academy of Sciences, Beijing 100101, China

^2^ Institute of Zoology, Academy of Sciences Republic of Uzbekistan, Bagishamol Str., 232b, Tashkent 100053, Uzbekistan

^3^ College of Life Sciences, University of Chinese Academy of Sciences, Beijing 100049, China

*** Corresponding authors** Gexia Qiao, [qiaogx@ioz.ac.cn](mailto:qiaogx@ioz.ac.cn); Jing Chen, chenjing@ioz.ac.cn.

**Supplementary Methods**

**Methods for Maximum Likelihood Phylogenetic Inference**

Based on the representative sequences of symbiont OTUs and the COI gene of aphids, the relatedness of *Buchnera* OTUs, secondary symbiont OTUs and *Mollitrichosiphum tenuicorpus* aphids were estimated using the maximum likelihood (ML) approach, respectively. *Mollitrichosiphum luchuanum*, *Mollitrichosiphum nigrum* and *Mollitrichosiphum rhusae* were used as outgroups in the phylogenetic tree of *M. tenuicorpus* aphids. GenBank accession numbers of sequences are listed in Table S1. All ML analyses were performed under the GTRCAT model with 1000 rapid bootstrapping replicates in RAxML v8.2.7 [1].

**References**

1. Stamatakis A (2014) RAxML version 8: a tool for phylogenetic analysis and post-analysis of large phylogenies. Bioinformatics 30:1312–1313. https://doi.org/10.1093/bioinformatics/btu033

**Table S1** Voucher information and GenBank accession numbers of aphid samples used in the present study

| Species | Voucher | Locality | Host plant | COI |
| --- | --- | --- | --- | --- |
| Ingroups^†^ |  |  |  |  |
| *Mollitrichosiphum tenuicorpus* Okajima | 13361 | Baoshan, Yunnan, China | *Meliosma rigida* | JQ926071^‡^ |
| *Mollitrichosiphum tenuicorpus* Okajima | 14421 | Mt. Wuyi, Fujian, China | *Castanea* sp. | JQ926070^‡^ |
| *Mollitrichosiphum tenuicorpus* Okajima | 14537 | Mt. Wuyi, Fujian, China | *Castanopsis sclerophylla* | JQ926069^‡^ |
| *Mollitrichosiphum tenuicorpus* Okajima | 15381 | Motuo, Tibet, China | *Alnus cremastogyne* | JQ926068^‡^ |
| *Mollitrichosiphum tenuicorpus* Okajima | 18506 | Lingshui, Hainan, China | *Cyclobalanopsis neglecta* | JF969321^‡^ |
| *Mollitrichosiphum tenuicorpus* Okajima | 18614 | Shixing, Guangdong, China | *Castanopsis carlesii* | JQ926066^‡^ |
| *Mollitrichosiphum tenuicorpus* Okajima | 18892 | Guilin, Guangxi, China | Fagaceae | JQ926065^‡^ |
| *Mollitrichosiphum tenuicorpus* Okajima | 19242 | Changjiang, Hainan, China | Fagaceae | JQ926064^‡^ |
| *Mollitrichosiphum tenuicorpus* Okajima | 19521 | Mt. Jianfengling, Hainan, China | *Quercus* sp. | JQ926063^‡^ |
| *Mollitrichosiphum tenuicorpus* Okajima | 20530 | Simao, Yunnan, China | *Castanopsis ferox* | JQ926062^‡^ |
| *Mollitrichosiphum tenuicorpus* Okajima | 20938 | Mt. Jianfengling, Hainan, China | *Castanopsis fabri* | JQ926060^‡^ |
| *Mollitrichosiphum tenuicorpus* Okajima | 22152 | Nanjing, Fujian, China | Unknown | JQ926059^‡^ |
| *Mollitrichosiphum tenuicorpus* Okajima | 22155 | Zhangzhou, Fujian, China | Unknown | JQ926058^‡^ |
| *Mollitrichosiphum tenuicorpus* Okajima | 22161 | Zhangzhou, Fujian, China | Unknown | JN644997^‡^ |
| *Mollitrichosiphum tenuicorpus* Okajima | 22166 | Zhangzhou, Fujian, China | Unknown | JQ926056^‡^ |
| *Mollitrichosiphum tenuicorpus* Okajima | 24067 | Ruili, Yunnan, China | *Castanopsis calathiformis* | JF969343^‡^ |
| *Mollitrichosiphum tenuicorpus* Okajima | 24074 | Ruili, Yunnan, China | *Castanopsis calathiformis* | JF969339^‡^ |
| *Mollitrichosiphum tenuicorpus* Okajima | 26029 | Huaping, Guangxi, China | *Castanopsis eyrei* | JN644999^‡^ |
| *Mollitrichosiphum tenuicorpus* Okajima | 26270 | Shangsi, Guangxi, China | Fagaceae | JQ418313^‡^ |
| *Mollitrichosiphum tenuicorpus* Okajima | 26892 | Mt. Wuyi, Fujian, China | *Castanopsis eyrei* | MZ073749 |
| *Mollitrichosiphum tenuicorpus* Okajima | 26906 | Jiangle, Fujian, China | Fagaceae | MT556455^‡^ |
| *Mollitrichosiphum tenuicorpus* Okajima | 27273 | Guilin, Guangxi, China | Unknown | MZ073750 |
| *Mollitrichosiphum tenuicorpus* Okajima | 31380 | Dujiangyan, Sichuan, China | *Castanopsis* sp. | MZ073751 |
| *Mollitrichosiphum tenuicorpus* Okajima | 38234 | Lishui, Zhejiang, China | *Lithocarpus glaber* | MZ073752 |
| *Mollitrichosiphum tenuicorpus* Okajima | 39115 | Nantou, Taiwan, China | *Castanopsis fargesii* | MZ073753 |
| *Mollitrichosiphum tenuicorpus* Okajima | 39171 | Nantou, Taiwan, China | *Castanopsis fargesii* | MZ073754 |
| Outgroups^†^ |  |  |  |  |
| *Mollitrichosiphum luchuanum* Takahashi | 18104 | Mt. Wuyi, Fujian, China | *Meliosma rigida* | JQ926105^‡^ |
| *Mollitrichosiphum nigrum* Zhang et Qiao | 14405 | Mt. Wuyi, Fujian, China | *Castanea* sp. | JQ926083^‡^ |
| *Mollitrichosiphum rhusae* Ghosh | 18508 | Mt. Diaoluo, Hainan, China | *Helicia hainanensis* | JQ926077^‡^ |

^†^Ingroups and outgroups refer to the ingroup and outgroup taxa used in ML analysis

^‡^Sequences downloaded from GenBank

**Table S3** Grouping information for the *Mollitrichosiphum tenuicorpus* samples used in the present study

| Aphid clades (3 groups) | Number of samples | Voucher |
| --- | --- | --- |
| Clade A | 1 | 15381 |
| Clade B | 3 | 13361, 24067, 24074 |
| Clade C | 22 | 14421, 14537, 18506, 18614, 18892, 19242, 19521, 20530, 20938, 22152, 22155, 22161, 22166, 26029, 26270, 26892, 26906, 27273, 31380, 38234, 39115, 39171 |
| Geographic region (12 groups) | Number of samples | Voucher |
| Fujian1 (FJ1) | 4 | 14421, 14537, 26892, 26906 |
| Fujian2 (FJ2) | 4 | 22152, 22155, 22161, 22166 |
| Guangdong (GD) | 1 | 18614 |
| Guangxi1 (GX1) | 3 | 18892, 27273, 26029 |
| Guangxi2 (GX2) | 1 | 26270 |
| Hainan (HI) | 4 | 18506, 19242, 19521, 20938 |
| Sichuan (SC) | 1 | 31380 |
| Taiwan (TW) | 2 | 39115, 39171 |
| Tibet (TB) | 1 | 15381 |
| Yunnan1 (YN1) | 3 | 13361, 24074, 24067 |
| Yunnan2 (YN2) | 1 | 20530 |
| Zhejiang (ZJ) | 1 | 38234 |
| Host plant (7 groups) | Number of samples | Voucher |
| *Alnus* | 1 | 15381 |
| *Castanea* | 1 | 14421 |
| *Castanopsis* | 11 | 14537, 18614, 20530, 20938, 24067, 24074, 26029, 26892, 31380, 39115, 39171 |
| *Cyclobalanopsis* | 1 | 18506 |
| *Lithocarpus* | 1 | 38234 |
| *Meliosma* | 1 | 13361 |
| *Quercus* | 1 | 19521 |

**Table S4** Relative abundance of the top 10 bacterial phyla, classes, orders, families and genera in *Mollitrichosiphum tenuicorpus*

| Phylum | Class | Order | Family | Genus |
| --- | --- | --- | --- | --- |
| Proteobacteria/98.85% | Gammaproteobacteria/96.66% | Enterobacteriales/96.28% | Enterobacteriaceae/96.26% | ***Buchnera***/83.62% |
| Bacteroidetes/0.36% | Alphaproteobacteria/1.85% | Rickettsiales/1.84% | Anaplasmataceae/1.41% | ***Arsenophonus***/10.52% |
| Firmicutes/0.30% | Betaproteobacteria/0.30% | Neisseriales/0.29% | Rickettsiaceae/0.41% | ***Wolbachia***/1.41% |
| Deinococcus-Thermus/0.09% | Flavobacteriia/0.29% | Flavobacteriales/0.29% | Neisseriaceae/0.29% | ***Hamiltonella***/0.77% |
| Tenericutes/0.04% | Bacilli/0.19% | Pasteurellales/0.16% | Blattabacteriaceae/0.29% | ***Rickettsia***/0.41% |
| Actinobacteria/0.04% | Deinococci/0.09% | Orbales/0.14% | Pasteurellaceae/0.16% | *Escherichia–Shigella*/0.39% |
| Fusobacteria/0.01% | Clostridia/0.09% | Bacillales/0.13% | Orbaceae/0.14% | *Neisseria*/0.27% |
|  | Bacteroidia/0.07% | Clostridiales/0.09% | Bacillaceae/0.12% | *Gilliamella*/0.14% |
|  | Mollicutes/0.04% | Thermales/0.08% | Thermaceae/0.08% | ***Serratia***/0.13% |
|  | Negativicutes/0.02% | Bacteroidales/0.07% | Ruminococcaceae/0.06% | *Haemophilus*/0.08% |

Aphid symbionts are indicated in bold

**Table S5** Alpha diversity of the bacterial, symbiont and secondary symbiont communities across *Mollitrichosiphum tenuicorpus* samples

| SampleID | Bacterial community | |  | Symbiont community | |  | Secondary symbiont community | |
| --- | --- | --- | --- | --- | --- | --- | --- | --- |
|  | Shannon | Simpson |  | Shannon | Simpson |  | Shannon | Simpson |
| 13361 | 0.296 | 0.223 |  | 0.268 | 0.210 |  | 0.482 | 0.402 |
| 14421 | 0.272 | 0.188 |  | 0.235 | 0.166 |  | 0.904 | 0.724 |
| 14537 | 0.470 | 0.434 |  | 0.448 | 0.426 |  | 0.371 | 0.328 |
| 15381 | 0.799 | 0.684 |  | 0.695 | 0.661 |  | 0.754 | 0.719 |
| 18506 | 0.396 | 0.280 |  | 0.260 | 0.221 |  | 0.406 | 0.316 |
| 18614 | 0.283 | 0.189 |  | 0.229 | 0.158 |  | 0.861 | 0.770 |
| 18892 | 0.150 | 0.096 |  | 0.142 | 0.092 |  | 0.792 | 0.706 |
| 19242 | 0.265 | 0.209 |  | 0.258 | 0.206 |  | 0.496 | 0.447 |
| 19521 | 0.228 | 0.148 |  | 0.175 | 0.121 |  | 0.923 | 0.808 |
| 20530 | 0.404 | 0.322 |  | 0.313 | 0.263 |  | 0.416 | 0.369 |
| 20938 | 0.146 | 0.093 |  | 0.103 | 0.068 |  | 0.495 | 0.436 |
| 22152 | 0.135 | 0.091 |  | 0.095 | 0.067 |  | 0.340 | 0.311 |
| 22155 | 0.117 | 0.077 |  | 0.085 | 0.061 |  | 0.242 | 0.190 |
| 22161 | 0.603 | 0.544 |  | 0.443 | 0.494 |  | 0.206 | 0.143 |
| 22166 | 0.571 | 0.504 |  | 0.437 | 0.459 |  | 0.289 | 0.219 |
| 24067 | 0.180 | 0.124 |  | 0.137 | 0.100 |  | 0.541 | 0.537 |
| 24074 | 0.448 | 0.317 |  | 0.308 | 0.254 |  | 0.589 | 0.589 |
| 26029 | 0.251 | 0.180 |  | 0.174 | 0.138 |  | 0.816 | 0.732 |
| 26270 | 0.520 | 0.416 |  | 0.392 | 0.366 |  | 0.569 | 0.611 |
| 26892 | 0.436 | 0.317 |  | 0.304 | 0.259 |  | 0.507 | 0.451 |
| 26906 | 0.327 | 0.248 |  | 0.225 | 0.184 |  | 0.546 | 0.470 |
| 27273 | 0.498 | 0.394 |  | 0.359 | 0.324 |  | 0.485 | 0.497 |
| 31380 | 0.660 | 0.575 |  | 0.522 | 0.531 |  | 0.462 | 0.542 |
| 38234 | 0.420 | 0.402 |  | 0.328 | 0.371 |  | 0.184 | 0.143 |
| 39115 | 0.738 | 0.646 |  | 0.624 | 0.619 |  | 0.532 | 0.528 |
| 39171 | 0.593 | 0.480 |  | 0.411 | 0.382 |  | 0.501 | 0.541 |

**Table S6** Separate effects of aphid genetic divergence and geography on microbial communities revealed by partial Mantel tests

| Beta diversity distance | Microbial community | Aphid genetic divergence | |  | Geography | |
| --- | --- | --- | --- | --- | --- | --- |
|  |  | r | *P* |  | r | *P* |
| Jaccard | Bacteria | 0.162 | 0.844 |  | 0.121 | 0.939 |
|  | Symbionts | 0.180 | 0.858 |  | 0.118 | 0.930 |
|  | Secondary symbionts | 0.328 | 0.992 |  | 0.111 | 0.947 |
| Bray–Curtis | Bacteria | 0.221 | 0.874 |  | 0.107 | 0.905 |
|  | Symbionts | 0.236 | 0.881 |  | 0.103 | 0.894 |
|  | Secondary symbionts | 0.358 | 0.993 |  | 0.097 | 0.918 |


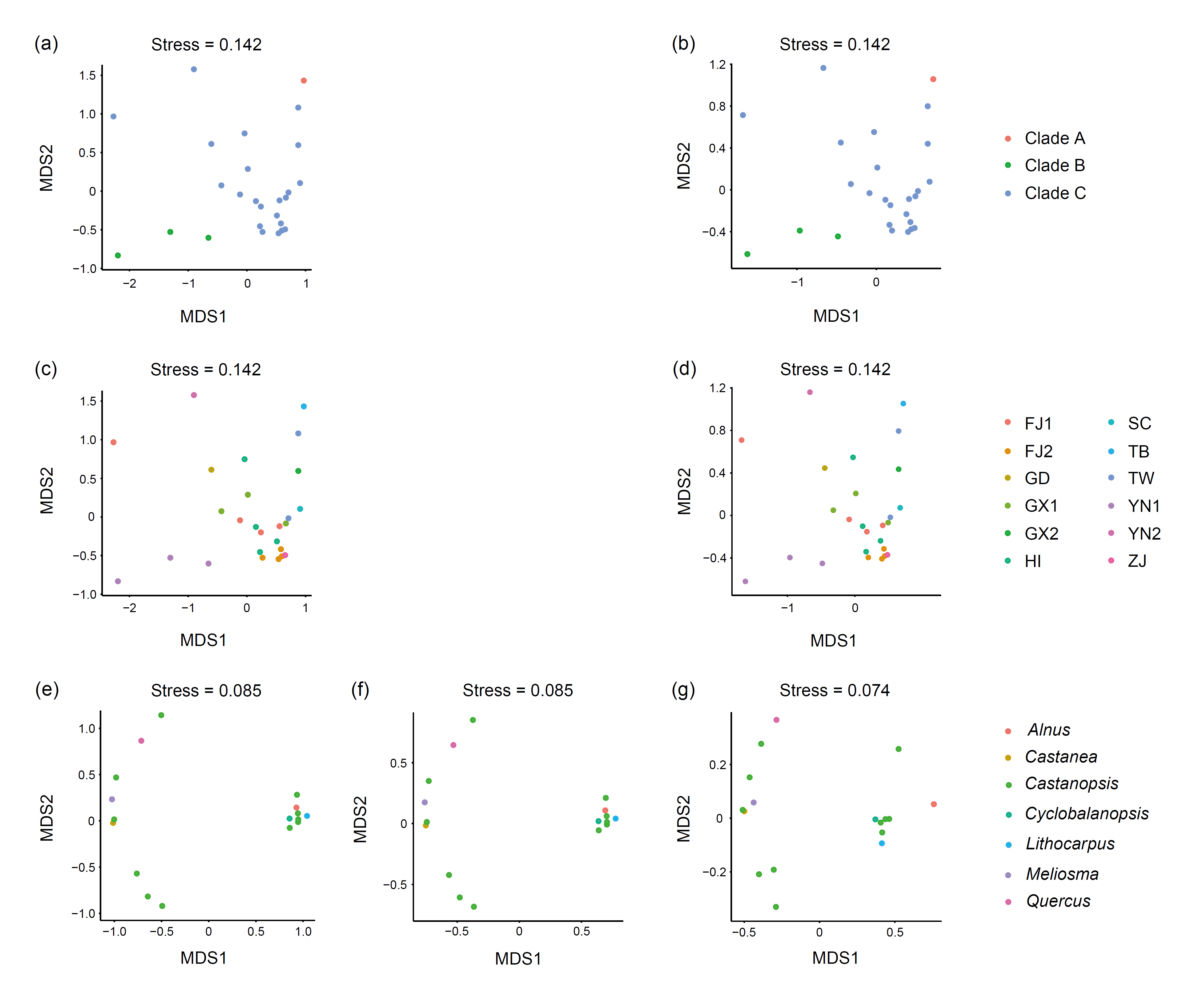


**Fig. S1** Nonmetric multidimensional scaling (NMDS) plots based on Jaccard (a, c, e) and Bray–Curtis distances (b, d, f, g) of symbiont (g) and secondary symbiont (a–f) communities (n ≥ 1). Samples are colored by aphid clades (a, b), geographic region (c, d) and host plant (e–g). The stress value indicates the goodness of fit between the NMDS representation and the data. The abbreviations are given in Table S3.


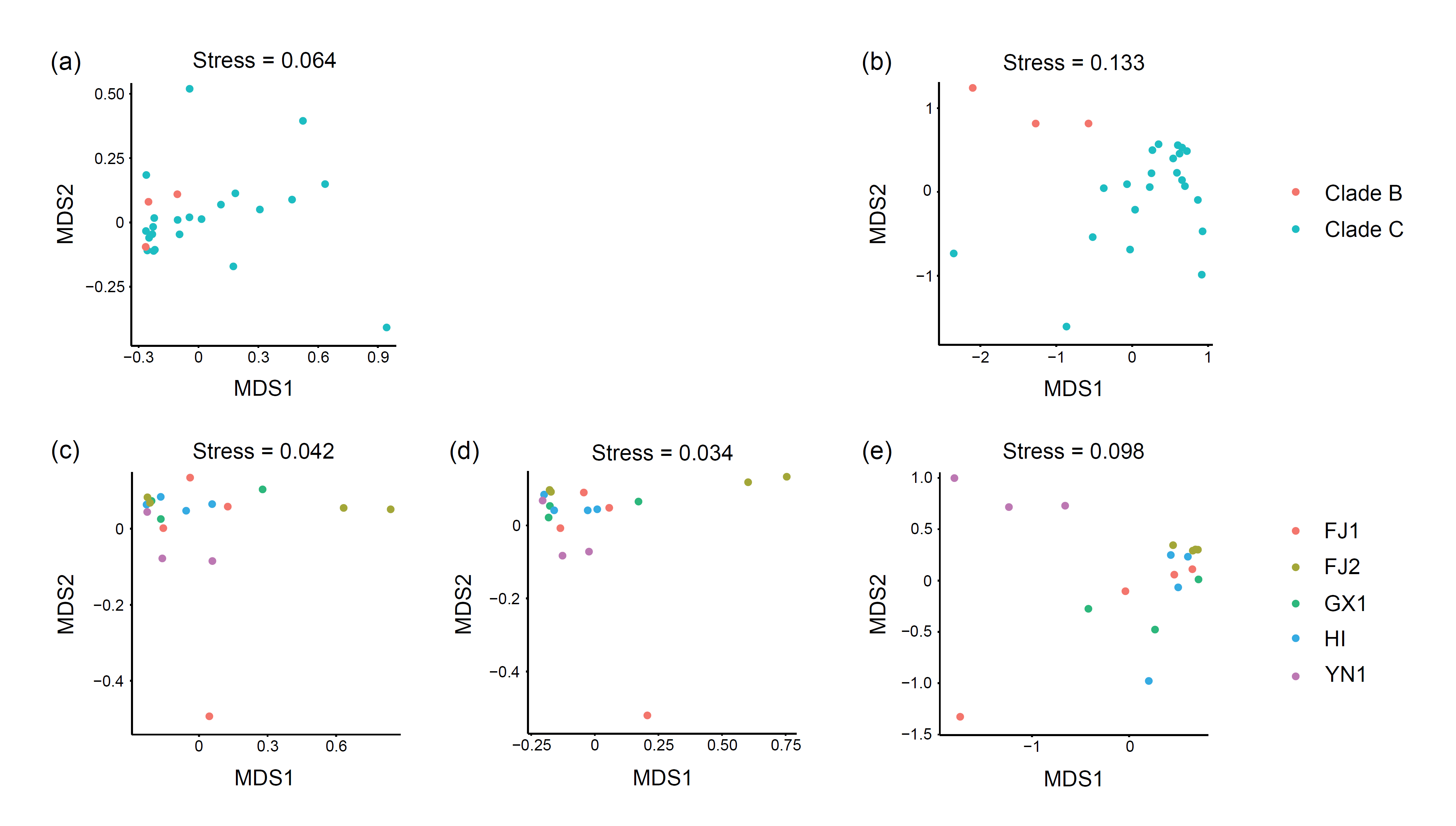
**Fig. S2** Nonmetric multidimensional scaling (NMDS) plots based on Jaccard distances of bacterial (c), symbiont (a, d) and secondary symbiont (b, e) communities (n ≥ 3). Samples are colored by aphid clades (a, b) and geographic region (c–e). The stress value indicates the goodness of fit between the NMDS representation and the data. The abbreviations are given in Table S3.


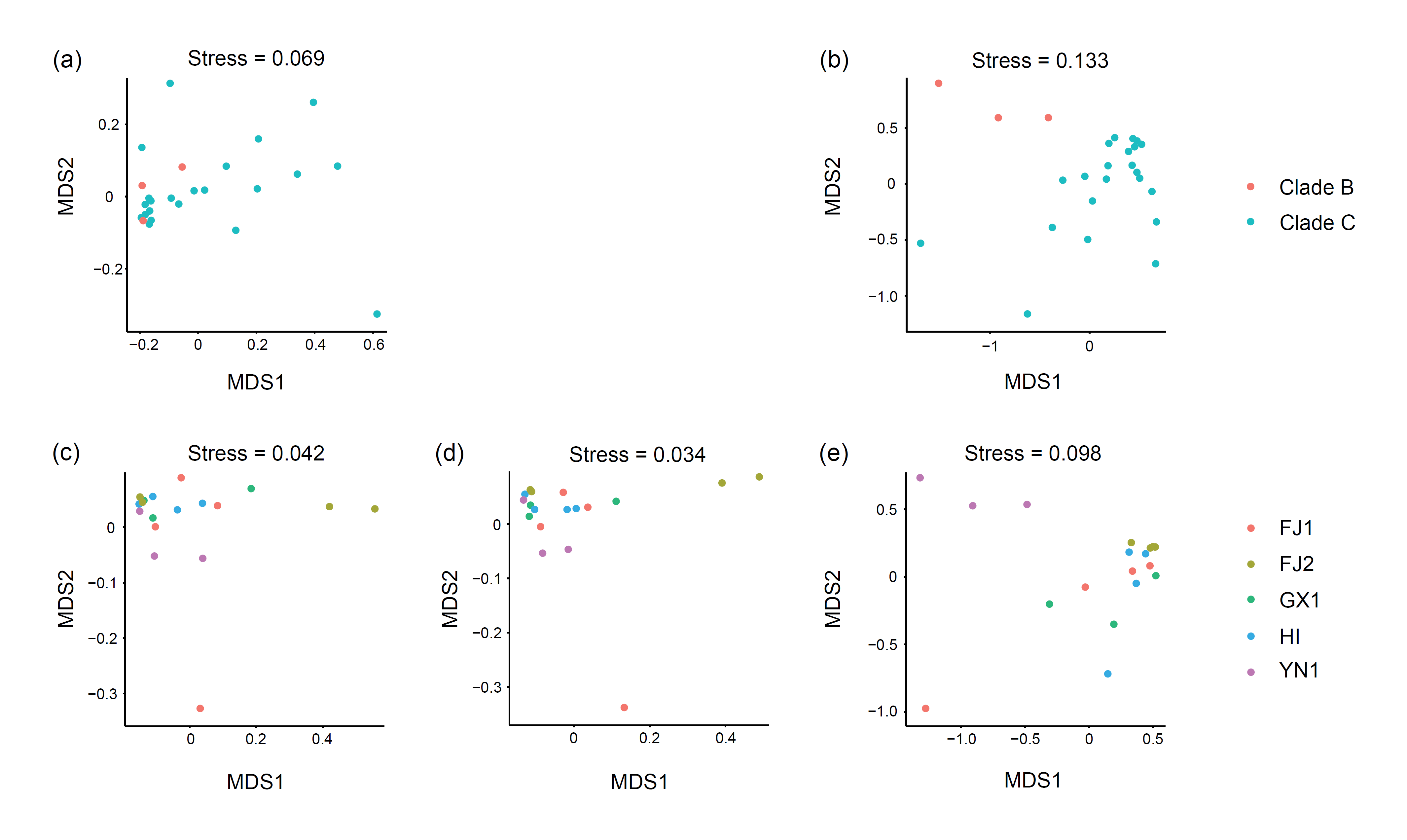
**Fig. S3** Nonmetric multidimensional scaling (NMDS) plots based on Bray–Curtis distances of bacterial (a, c), symbiont (d) and secondary symbiont (b, e) communities (n ≥ 3). Samples are colored by aphid clades (a, b) and geographic region (c–e). The stress value indicates the goodness of fit between the NMDS representation and the data. The abbreviations are given in Table S3.


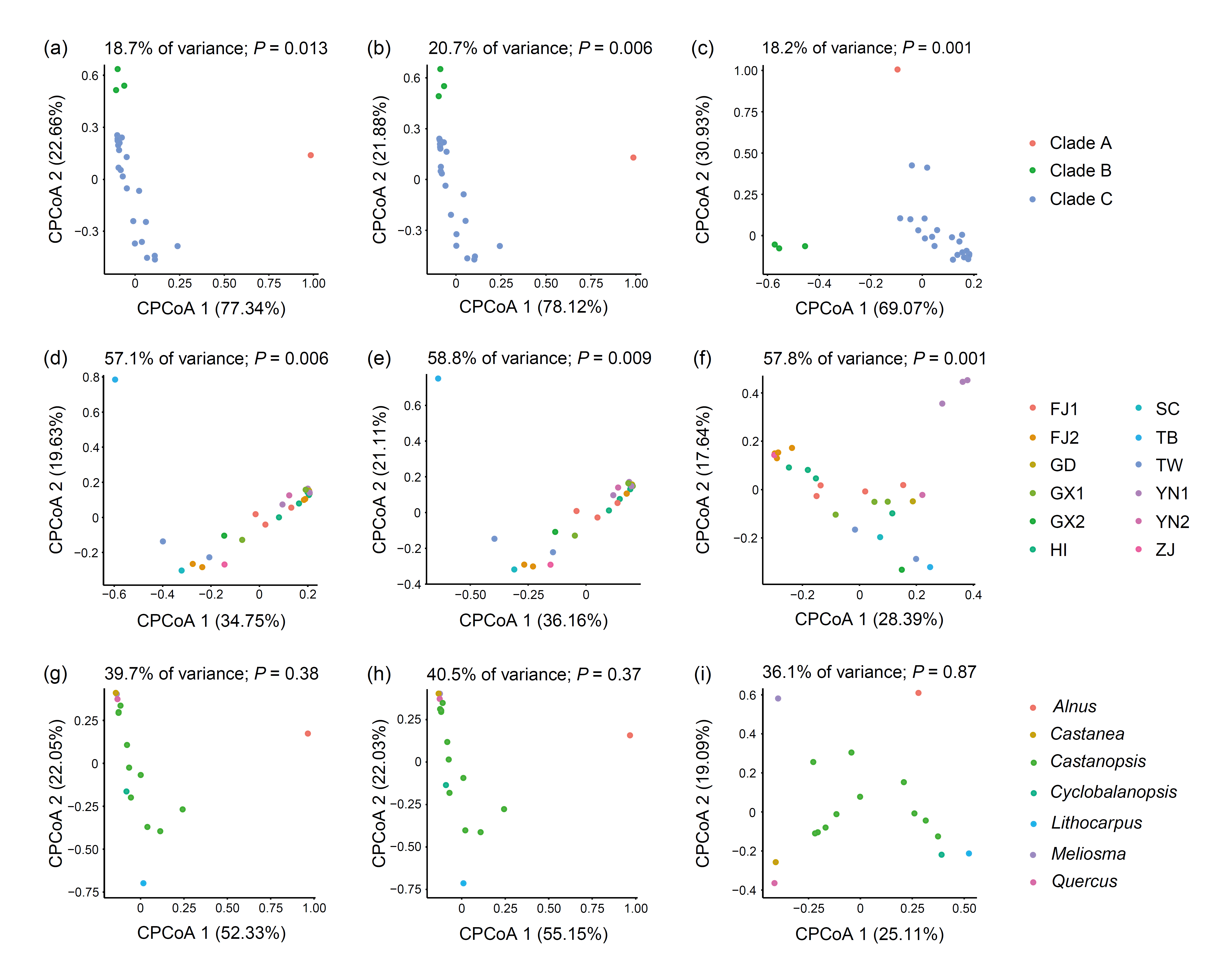
**Fig. S4** Structural segregation using constrained principal coordinate analyses (cPCoA) of Jaccard distances of bacterial (a, d, g), symbiont (b, e, h) and secondary symbiont (c, f, i) communities (n ≥ 1). Plots are structured by aphid clades (a–c), geographic region (d–f) and host plant (g–i). The overall variation explained by the constrained factor is displayed at the top of each plot. The percent variation shown on each axis refers to the fraction of the total variance explained by the projection. The abbreviations are given in Table S3.


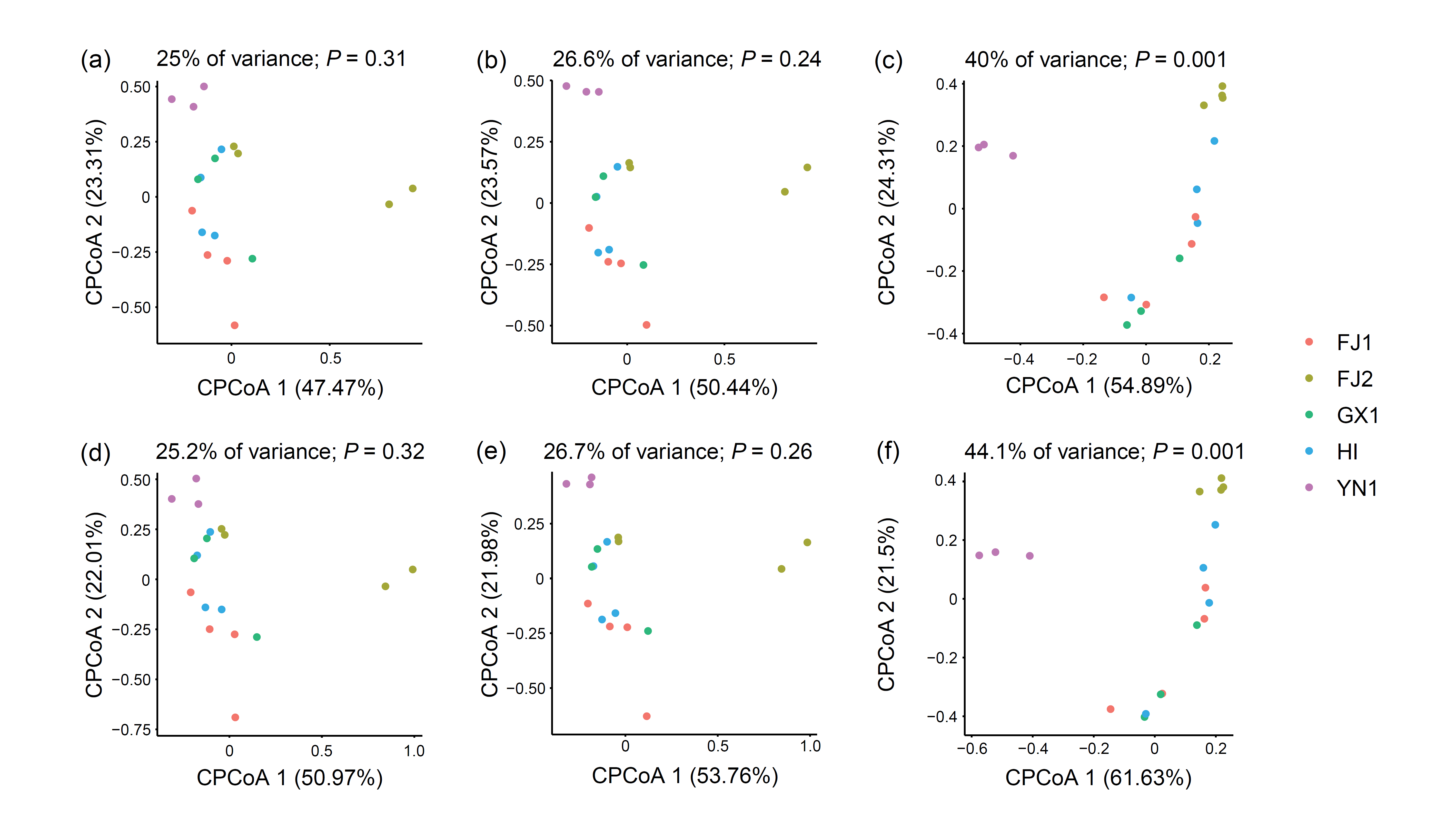
**Fig. S5** Structural segregation using constrained principal coordinate analyses (cPCoA) of Jaccard (a, b, c) and Bray–Curtis (d, e, f) distances of bacterial (a, d), symbiont (b, e) and secondary symbiont (c, f) communities (n ≥ 3). Plots are structured by geographic region. The overall variation explained by the constrained factor is displayed at the top of each plot. The percent variation shown on each axis refers to the fraction of the total variance explained by the projection. The abbreviations are given in Table S3.


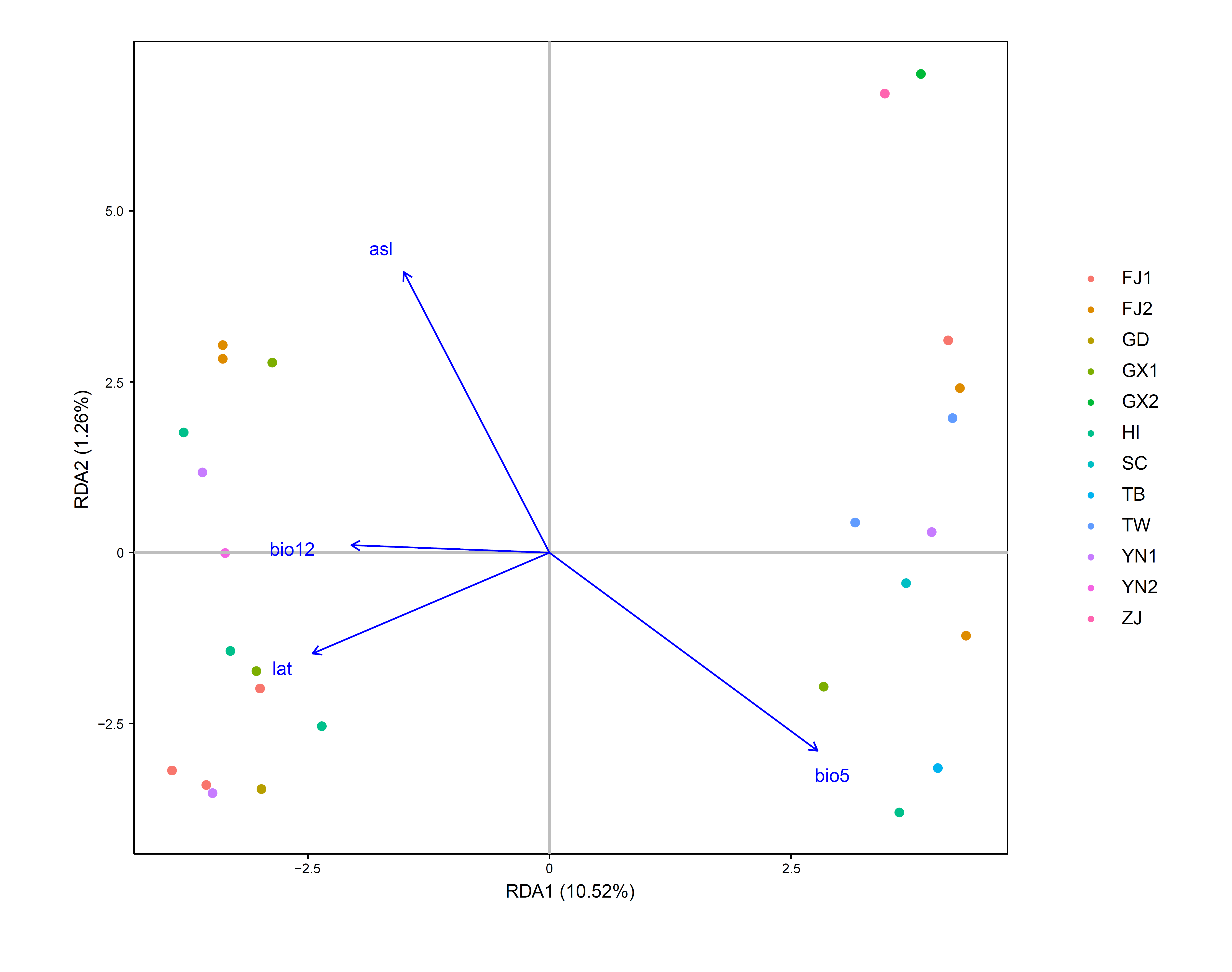


**Fig. S6** Redundancy analysis (RDA) ordination plot of environmental variables and bacterial communities in *Mollitrichosiphum tenuicorpus*. The abbreviations of geographic regions are provided in Table S3.
